# Providence Asylum for the Insane and Inebriate

**Published:** 1887-08

**Authors:** 


### *PROVIDENCE ASYLUM FOR THE INSANE AND INEBRIATE.*

As will be seen by reference to the advertisement on another page, the Providence Asylum has been entirely refitted and the staff so reorganized as to meet the demands upon it. The institution is able now to treat any and all cases of insanity and inebriety. The wards are small and thus a great number of patients are not brought in contact with each other, yet they are large enough to afford every comfort to the patients. The rooms and buildings are thoroughly heated by steam, making it most comfortable in winter. The location of the building on the highest point of land in Buffalo, and situated in the middle of a large tract of land, makes it delightfully cool in summer.

Every hall in the house is under charge of a Sister of Charity, who has had years of experience in the care of this class of cases, and whose devotion to the sick is proverbial. It has a medical staff consisting of men who have had experience in this class of cases.

The medical supervision, which for many years was conducted by the late Dr. Ring, has fallen naturally to Dr. Crego, who attended with him for a year, and who has had several years' experience in the Buffalo State Asylum. He is ably seconded by Dr. Wm. G. Ring, who assisted more or less for three years in the asylum. They are assisted by two young men, Dr. Lewis and Dr. Congdon, who have had liberal educations in medicine, and studied nervous and mental diseases under the instruction of Dr. Crego in the Medical Department of Niagara University. The public have already taken a deeper interest in the institution, as shown by the increased number of admissions during last year. The Sisters, as well as their friends and patients, are rejoiced at the great number of recoveries during the last year. The home life, freedom from curious visitors, the quiet of the place, and its strictly private character, are the advantages claimed for the institution. The staff is as follows:

Thomas Lothrop, M. D., Edward C. W. O'Brien, M. D., Conrad Diehl, M. D., consulting physicians; Floyd S. Crego, M. D., 141 Delaware avenue, senior attending physician, and chief of staff; William G. Ring, M. D., 364 Niagara street, junior attending physician; George W. T. Lewis, M. D., senior assistant, Linwood avenue and Main street; Charles G. Congdon, M. D., junior assistant, 1046 Jefferson street.

---

Why is it that operations about the throat seem to lead the professional mind to a desire for newspaper notoriety? It is because the windpipe supplies a connection to the idea of "blowing one's own horn?" However it may be explained, events would seem to indicate some such conclusion. Some time ago we had successive reports of one operation on a man's larynx, and the public were duly impressed with the extraordinary nature of the operation and the superlative skill of the operator. A few days ago, under the astounding title of "A Modern Lazarus," we find a column of a newspaper devoted to a graphic and detailed account of a laryngotomy, under circumstances which made the operation creditable to the surgeon it is true, but reported in a way which has justly met with reproof and condemnation by the regular profession.

